# Detection of HER2 from Haematoxylin-Eosin Slides Through a Cascade of Deep Learning Classifiers via Multi-Instance Learning

**DOI:** 10.3390/jimaging6090082

**Published:** 2020-08-23

**Authors:** David La Barbera, António Polónia, Kevin Roitero, Eduardo Conde-Sousa, Vincenzo Della Mea

**Affiliations:** 1 Department of Mathematics, Computer Science and Physics, University of Udine, 33100 Udine, Italy; labarbera.david@spes.uniud.it (D.L.B.); roitero.kevin@spes.uniud.it (K.R.); 2 Department of Pathology, Ipatimup Diagnostics, Institute of Molecular Pathology and Immunology, University of Porto, 4169-007 Porto, Portugal; apolonia@ipatimup.pt; 3 i3S—Instituto de Investigação e Inovação em Saúde, Universidade do Porto, 4169-007 Porto, Portugal; econdesousa@gmail.com; 4 INEB—Instituto de Engenharia Biomédica, Universidade do Porto, 4169-007 Porto, Portugal

**Keywords:** digital pathology, whole slide image processing, multiple instance learning, convolutional neural networks, deep learning classification, HER2

## Abstract

Breast cancer is the most frequently diagnosed cancer in woman. The correct identification of the HER2 receptor is a matter of major importance when dealing with breast cancer: an over-expression of HER2 is associated with aggressive clinical behaviour; moreover, HER2 targeted therapy results in a significant improvement in the overall survival rate. In this work, we employ a pipeline based on a cascade of deep neural network classifiers and multi-instance learning to detect the presence of HER2 from Haematoxylin–Eosin slides, which partly mimics the pathologist’s behaviour by first recognizing cancer and then evaluating HER2. Our results show that the proposed system presents a good overall effectiveness. Furthermore, the system design is prone to further improvements that can be easily deployed in order to increase the effectiveness score.

## 1. Introduction

Breast cancer is the most frequently diagnosed cancer in women. As shown by Siegel et al. [1], breast cancer in 2020 is expected to account for 30% of female cancer in United States. The diagnosis of breast cancer, including its morphological subtypes, is that was carried out by a pathologist by examining a microscope histologic slide obtained from a breast biopsy or from a surgical sample. The slide is stained with a two-colors staining, called Haematoxylin-Eosin (HE), which enables the recognition of morphological features of the cancer. Haematoxylin-Eosin (HE) is universally used for histologic diagnosis. In addition to that, further sections are prepared and stained with immunohistochemical techniques (IHC) to evidentiate the expression of specific proteins. In the case of breast cancer, routine examination includes oestrogen and progesterone receptors, MIB2, and HER2. Visually, HE-stained slides show blue (for nuclei) and pink (for cytoplasm), while IHC slides are brown where the investigated protein is expressed, and counterstained in blue by means of haematoxylin.

According to current guidelines of the College of American Pathologists for HER2 testing in breast cancer [2], Human Epidermal growth factor Receptor 2 (HER2) quantification must be routinely tested, in invasive breast cancer, recurrences, and metastases. HER2 is a trans-membrane protein receptor with tyrosine kinase activity; studies have shown that HER2 is amplified and/or over-expressed in about 25% of breast cancer cases [3]. The over-expression and/or amplification of the HER2 receptor have been associated with aggressive clinical behaviour; nevertheless, the accurate assessment of the HER2 receptor has proven to be essential for identifying breast cancer patients who will benefit from HER2-targeted therapy [4]. It has been demonstrated by many clinical trials that the HER2-targeted therapy (both when administrated during and/or after chemotherapy) results in a significant improvement in both disease-free and overall survival in patients who have shown either an amplification or over-expression of the HER2 receptor [4]. Consequently, the correct identification of the HER2 receptor lead to select patients who are expected to benefit from the targeted therapy; this makes HER2 a helpful marker for the therapy decision making process.

Many researchers tried to develop IHC techniques that are able to identify HER2 with a sufficient precision and therefore be able to help the pathologist with the characterization of breast cancer [5,6,7,8,9,10,11,12,13]. The current approach to HER2 testing consists in cutting an additional section for the IHC evaluation of the positivity, negativity, or uncertain status of HER2 expression. In uncertain cases, a further examination has to be done based on In-Situ Hybridization, increasing costs and time. This, in addition to the HE stained slide, which is always prepared because needed for morphological diagnosis of cancer and of its subtypes. In the context of digital pathology, there is an increasing interest in developing Deep Learning (DL) techniques that can support the decision making process of the pathologist, especially when dealing with cancer related topics [10]. The practical use of deep learning in the context of breast cancer diseases is mainly due the performance achieved by deep learning algorithms over the last decade in the field of image recognition [14,15,16].

The recent HEROHE challenge proposed a peculiar research question: is it possible to identify HER2 from Hematoxylin–Eosin images of invasive breast cancer? In case of success, this would allow obtaining HER2 status without the corresponding IHC image, thus reducing time and costs. In fact, it is known that HER2 positivity is associated with aggressiveness of the tumour, which, in turn, is associated with certain morphological features, such as high histological grade. Thus, the idea is to exploit such morphological features as a proxy for HER2 status. For this, the Challenge organizers provided a set of 400 digitized haematoxylin-eosin slides accompanied by their HER2 status, and subsequently 150 slides for testing.

The objective of our study is the same behind the HEROHE challenge: to find a method to recognize the therapeutically useful HER2 status from the morphological aspects of the breast cancer only, as recognisable in the HE slides that are already used for diagnosis.

More in detail, our approach is based on deep learning algorithms employed in the setting of image recognition and Multiple Instance Learning (MIL) [17,18,19,20,21].

Digitized microscope slides, normally called digital slides or Whole Slide Images (WSI), correspond to huge scanned images that are difficult to process with standard Machine Learning (ML) or Deep Learning (DL) approaches due to hardware limitations [22]. A technique that is used to overcome such limitations is represented by MIL [23,24]. Opposed to the classical ML and DL classification setting where the learning algorithm receives in input a set of individually labelled instances, in MIL the learning algorithm receives a set of labelled instances (in our case, each instance is a WSI), each of these containing many unlabelled instances (in our case, the WSI is divided into tiles). Subsequently, the final class for the instance (i.e., the WSI) is decided upon the predicted classes of the unlabelled instances (i.e., the tiles). For example, in the simplest case, the main instance may be labelled as negative if all its unlabelled instances in it are labelled as negative negative [25].

We now detail some work related to the work of this paper.

MIL has been proven to be effective in the setting of image recognition and, in particular, in the context of cancer detection: Campanella et al. [26] used MIL to tackle the problem of prostate cancer detection, basal cell carcinoma, and breast cancer metastases to axillary lymph nodes. Their approach resulted in areas under the ROC curve (AUC) above 0.98 for the cancer types considered in the study.

MIL has also been used in the setting of breast cancer image detection. P J et al. [27] conducted experiments on a public dataset of biopsy images of benign and malignant breast cancers; according to their findings, MIL provides better results than state-of-the-art approaches in the setting of the detection of breast cancer in images. A more relevant work to our setting is the study conducted by Couture et al. [28], which investigated a method to predict cancer subtypes from histologic images. Couture et al. used MIL to model the problem and account for intra-cancer variations in cancer subtypes, improving the correct classification of cancer subtypes with reference to the state of the art approaches, and providing also insights about cancer heterogeneity.

Other researchers tried to detect HER2 from HER2 stained digital slides, using ML or not ML methods [29,30,31,32,33,34]; we do not detail their methodology, since they are out of scope for this work.

Thus, our specific contribution is a pipeline based on a cascade of deep neural network classifiers and multi-instance learning, which partly mimics the pathologist’ behaviour by first recognizing cancer and then evaluating HER2, and able to currently provide state-of-the-art performance for the novel problem of detecting HER2 from Haematoxylin–Eosin slides.

## 2. Materials and Methods

### 2.1. Dataset and Materials

**Figure 1:**
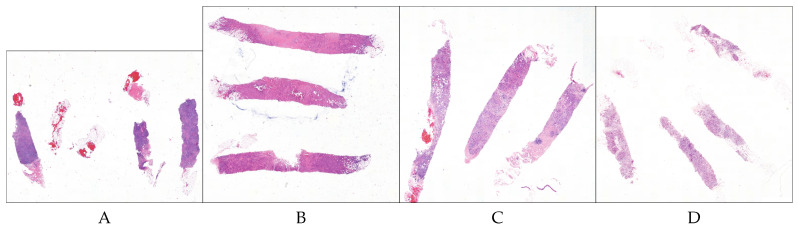
Example of tiles from the HEROHE dataset. The left-most two slides (**A**,**B**) have been labelled as positive for HER2; The right-most two slides (**C**,**D**) have been labelled as negative.

In our study, we used the dataset made available from the Instituto de Investigação and Inovação em Saúde and Instituto de Engenharia de Sistemas e Computadores, Tecnologia e Ciência from Porto University in Porto, Portugal, who together organized the HEROHE Grand Challenge for ECDP2020 (see https://ecdp2020.grand-challenge.org). The classification of this dataset used the latest American Society of Clinical Oncology/College of American Pathologists (ASCO/CAP) classification of breast cancer (Focused Update 2018) [2]. The HEROHE training dataset is composed of 144 positive slides (i.e., WSI of cases which are labelled as HER2 positive) and 216 negative slides (i.e., WSI of cases that are labelled as HER2 negative). Figure 1 shows an example of positive and negative slides present in the dataset.

After implementing the proposed solution (as detailed in the next section), we tested it on the HEROHE Grand Challenge test set that is composed of 60 positive and 90 negative slides. The training, validation, and testing phase has been carried out using either a Titan XP GPU or using the Google Cloud Platform. The proposed solution has been implemented on the fast.ai Framework. Due to the nature of the data used in this study, we cannot publicly release the images used to train our algorithms. However, we release all of the code used to conduct the experiments as well as the trained models (Supplementary Materials).

### 2.2. Our Approach: A Cascade of Deep Learning Classifiers

The idea behind this work is to build a system that mimics the actions a pathologist performs when detecting the presence of the HER2 receptor. The actions include to divide each slide into tiles under the “divide et impera” principle, filter the tiles in order to keep only the most informative ones for the purpose of HER2 detection (i.e., only the tiles containing cancer), check for the presence of HER2 into the individual tiles, and finally aggregate the tile information at the slide level to perform the decision whether the whole slide expresses or not the HER2 receptor.

**Figure 2:**
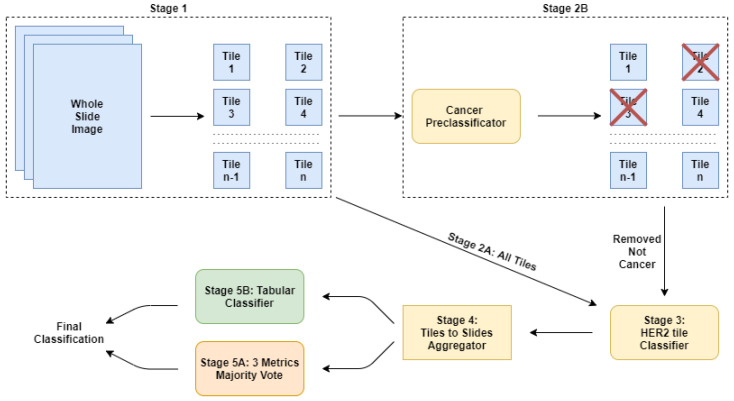
Graphical overview of the multi-stage deep learning pipeline.

To this aim, we developed a multi-stage pipeline as graphically represented in Figure 2. We considered a multi-stage system because this architectural design allowed us to try different techniques (or different variants of a single technique) for each stage, and then deploy to the final version of the system using only the most effective combination of stages. Furthermore, a multi-stage pipeline is the ideal scenario to test different approaches and compare single techniques at different levels of the pipeline. The pipeline we implemented is composed, as follows: the first stage extracts the tiles (Section 2.2.1), second stage considers either all tiles (Section 2.2.2), or a subset of them (Section 2.2.3), third stage details the main classifier architecture (Section 2.2.4), fourth stage formalize the features considered (Section 2.2.5), and fifth stage consider the post-training aggregation by means of either majority voting (Section 2.2.6) or a Tabular learner (Section 2.2.7). In the following sections, we discuss the role of each stage in detail.

#### 2.2.1. Stage 1: The Tiles Extractor

The first stage of our pipeline is devoted to extract the tiles from every WSI present in the dataset. We consider each tile to be an instance necessary? (using the DL nomenclature) of our problem formulation. Slides provided by the HEROHE challenge were scanned at 40× magnification (which corresponds to a pixel size of 0.25 μm/pixel), with a 3DHistech Pannoramic 1000 scanner, and stored in their proprietary format. For the present work, slides were subsampled to 20× magnification (which has proven to reduce the computational complexity, but not decrease the overall accuracy with reference to the 40× magnification [26]) and converted to TIFF format. Therefore, the pixel size of the used slides corresponds to 0.50 μm/pixel.

**Figure 3:**
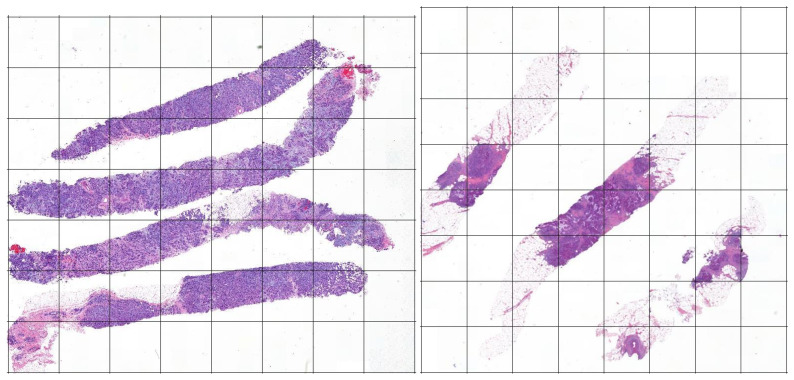
An example of slides from our dataset in which we applied a rectangular grid (shown with the continuous black lines) to show the division of a slide in tiles.

After such process, each slide is then decomposed into squares (i.e., the tiles) of 512×512 pixels, with no stride. Figure 3 shows an example of the decomposition of two slides into tiles. Note that the slides in the dataset might have different dimensions, thus each of them is decomposed in a variable number of tiles.

Subsequently, in order to increase the overall accuracy model and preserve salient features [35], we dropped the tiles that have an average grey-scale value (i.e., the pixel value that represents the brightness of the pixel; the lower the value, the darker the pixel) greater than 0.8. This allows to discard background slides, including those presenting very small amounts of tissue.

After this stage, we considered two different approaches: the former consists in passing forward to the next stage all of the tiles; the latter in considering only an informative subset of tiles. Such approaches are detailed respectively in Section 2.2.2 and  Section 2.2.3.

#### 2.2.2. Stage 2A: Using All the Tiles

This stage simply consists in keeping all the tiles, without discarding any, and forwarding them without any modification to the next stage. The rationale behind this stage is that by maintaining all of the tiles we do not discard any potentially useful information, and we delegate to (the classifier of) the next stages the task to discern between relevant and not relevant information (i.e., noise) for all of the tiles considered.

#### 2.2.3. Stage 2B: Using a Subset of Tiles

This stage takes in input all of the tiles from Stage 1 (see Section 2.2.1) and filters them, under the rationale that a subset of tiles is more informative for the model than the whole set of (potentially noisy) tiles. Moreover, this process simulates the real case scenario; in fact, when looking for the presence of HER2 in slides, the pathologist focus the search only in the part of slides (similar to our tiles) containing cancer, and more specifically in the tiles containing invasive cancer. One additional reason is that HER2 might also be expressed outside the cancer tissue, in normal ducts, or as extracellular accumulation, or also as staining artifacts. While we might expect that DL is able to cope with these situations too, we did not want to expose the network to spurious data, or at least to reduce the amount of noise. We perform a pre-classification task, with the purpose of discerning between the tiles that contain cancer and those that do not, in order to filter the input tiles. To this aim, we developed a model based on a Convolutional Neural Network (CNN), relying on the model detailed by Della Mea and Pilutti [36], which is based on the densenet-201 architecture [37,38,39] pretrained on the ImageNet dataset [40]. However, the HEROHE data set did not include annotations for identifying cancer areas inside the slides, which may as well contain normal tissue or tissue representing other not tumoral diseases. Thus, although not optimal, we had to exploit a different set of training images. For this aim, we fine-tuned the model on 400 images from the BACH Challenge Part A (see https://iciar2018-challenge.grand-challenge.org/) [41] dataset. These images are 2048 × 1536; by means of SlideJ [42] we extracted tiles of 512 × 512 size, with a stride of 256, obtaining 35 tiles per image. Thus, we trained for three epochs to fine-tune the last layer, plus three epochs for the whole network. We applied the default fast.ai data augmentation transforms, including random mixtures of flips, rotations, moderate zoom (up to 1.1×), warping, luminosity, and contrast variations. We collapsed the original labels (i.e., “normal”, “benign”, “in situ carcinoma”, and “invasive carcinoma”, 100 images for each class, corresponding to 3500 tiles) into two binary categories, as follows: we mapped the original labels “normal” and “benign” into the label “no cancer”, and the labels “in situ carcinoma” and “invasive carcinoma” into the label “cancer”. Note that we also consider tiles that contain in-situ cancer because, at high resolution, it would be impossible to distinguish them from the ones with invasive cancer.

**Figure 4:**
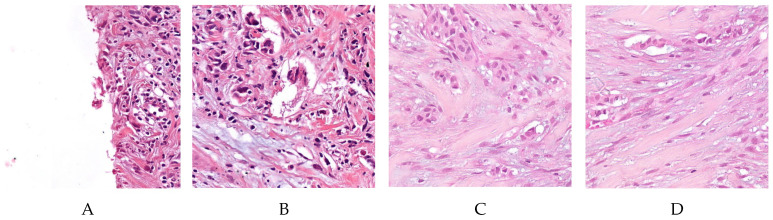
Tiles identified by the cancer classifier as: positive (first two tiles, tile (**A**) is a true positive, tile (**B**) is a false positive), and negative (last two tiles, tile (**C**) is a true negative, tile (**D**) is a false negative).

After the training phase, we fed all of the tiles considered in this work to the fine-tuned classifier, and we removed the tiles classified as not containing cancer. Overall, we fed the model with 90.068 test tiles, and we removed 29.429 tiles (32.68%), maintaining 60.639 (67.32%). On average, we removed the 29.27% of tiles from each slide; more in detail, on average, we removed 32.02% of tiles from negative slides and 25.16% from positive slides. Figure 4 shows an example of the classification outcome in term of slides.

#### 2.2.4. Stage 3: The Main Classifier

**Figure 5:**
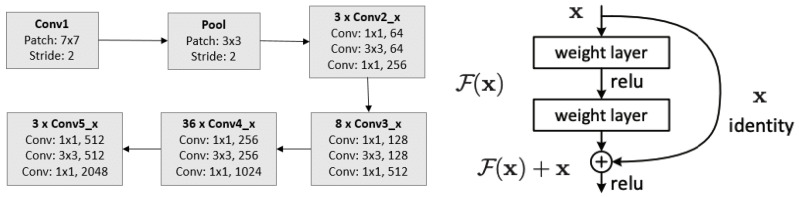
The basic architecture of Resnet152 (**left**, from [48]), and the building block for the residual learning (**right**, from [43]).

After the second stage, we trained the main classifier, either with all tiles (from stage 2A) or with the subset of tiles labelled as containing cancer (from stage 2B). Our main architecture is as follows. We trained a classifier based on the ResNet152 architecture [43], a well-known architecture for image recognition [43,44,45,46] based on the concept of residual learning [43]. Figure 5 provides a schema of the model architecture and the block for residual learning. We fed the classifier each tile with the associated HER2 status. We did not expect to obtain high accuracy, because in a positive slide HER2 is not expressed everywhere; on the other side, in a negative slide, we expect HER2 not to be expressed, although it is known that it could also be present in normal tissue, which is one reason why we wanted to discard not cancer tiles. We trained the classifier using the HEROHE training set for three epochs while using precision as loss function. For data augmentation, we applied the same transforms as the first classifier. Learning rate has been set according the One-cycle policy developed by L.Smith [47].

**Figure 6:**
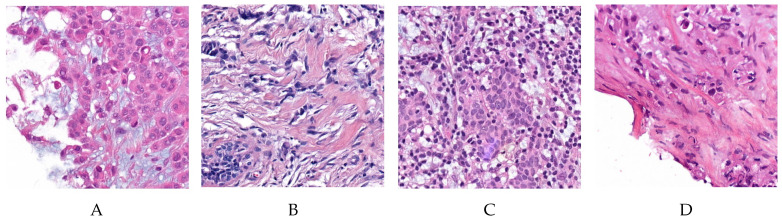
Tiles identified by the HER2 classifier as positive (first two tiles, tile (**A**) is a true positive, tile (**B**) is a false positive), and negative (last two tiles, tile (**C**) is a true negative, and tile (**D**) is a false negative).

Figure 6 shows an example of the classification outcome in terms of tiles.

#### 2.2.5. Stage 4: Computing the Features

The classifier detailed in the previous stage returns as output, for each test instance, the probability that the test instance belongs to a given class, for all of the classes. In our setting, the classifier returns a probability value in the [0,1] range that denotes the probability of each tile in be positive for the HER2 receptor. Those are the probabilities we will consider in this section. After training our main classifier to determine for each tile its probability of containing the HER2 receptor, we aggregated the results at the tile level to determine the overall classification outcome, which correspond to assess whether the whole slide contains or not the HER2 receptor. To this aim, we used two strategies detailed in the following, which take advantage of the features described in this section.

Suppose that we have S slides, and that each slide is divided into T tiles. We denote with $p_{i}^{j}$ is the probability of the *i*-th tile (belonging to the *j*-th slide) to be tested positive for HER2.

Subsequently, in the first case we compute the binary positivity $P_{1}\left(s_{i}\right)$ of the slide $s_{i}$ by considering the simple arithmetic average of the probabilities $p_{i}^{j}$ for all of the tiles; formally:

$$P_{1}\left(s_{i}\right)=\left\{\begin{array}{cc} 1 & \mathrm{if}\;\frac{1}{T}\sum_{i=1}^{T}p_{i}^{j}\geq 0.5,\\ 0 & \mathrm{otherwise}. \end{array}\right.$$

In the second case, we compute the binary positivity $P_{2}\left(s_{i}\right)$ of the slide $s_{i}$ by considering the simple arithmetic average of the probabilities $p_{i}^{j}$ for tiles having probability $p_{i}^{j}\geq 0.5.$; formally:

$$P_{2}\left(s_{i}\right)=\left\{\begin{array}{cc} 1 & \mathrm{if}\;\frac{1}{T}\sum_{i=1}^{T}p_{i}^{j}\cdot G\left(p_{i}^{j}\right)\geq 0.66,\\ 0 & \mathrm{otherwise}, \end{array}\right.$$

where

$$G\left(p_{i}^{j}\right)=\left\{\begin{array}{cc} 1 & \mathrm{if}\;p_{i}^{j}\geq 0.5,\\ 0 & \mathrm{otherwise}. \end{array}\right.$$

In the third case, we compute the binary positivity $P_{3}\left(s_{i}\right)$ of the slide $s_{i}$ by considering the slide positive if at least the 35% of its tiles have a probability $p_{i}^{j}\geq 0.5$; formally:

$$P_{3}\left(s_{i}\right)=\left\{\begin{array}{cc} 1 & \mathrm{if}\;\frac{1}{T}\sum_{i=1}^{T}G\left(p_{i}^{j}\right)\geq 0.35,\\ 0 & \mathrm{otherwise}. \end{array}\right.$$

Apart from the three positivity indices $P_{1}\left(s_{i}\right)$, $P_{2}\left(s_{i}\right)$, and $P_{3}\left(s_{i}\right)$ of each slide, we consider, as additional set of features, the frequency of the probabilities $p_{i}^{j}$ for each slide, for each interval [h,h+0.1), h∈{0.5,0.6,0.7,0.8,0.9}; formally,

$$F_{h}\left(s_{i}\right)=\frac{1}{T}\sum_{i=1}^{T}C\left(p_{i}^{j},h\right),$$

where

$$C\left(p_{i}^{j},h\right)=\left\{\begin{array}{cc} 1 & \mathrm{if}\;p_{i}^{j}\in \left[h,h+0.1\right),\\ 0 & \mathrm{otherwise}. \end{array}\right.$$

After computing the positivity indices $P_{1}\left(s_{i}\right)$, $P_{2}\left(s_{i}\right)$, and $P_{3}\left(s_{i}\right)$, and the set of features $F_{h}\left(s_{i}\right)$, h∈{0.5,0.6,0.7,0.8,0.9}, we aggregate such indices to compute the overall positivity of the *i*-th slide. To do so, we adopt two strategies, detailed in the following.

#### 2.2.6. Stage 5A: Post-Trainer Aggregation Using Majority Vote

The first strategy we adopt to compute the overall positivity of each slide is to simply compute the majority vote of the $P_{1}\left(s_{i}\right)$, $P_{2}\left(s_{i}\right)$, and $P_{3}\left(s_{i}\right)$ indices. In this way, we assume that all of the features are equally informative for the final decision.

#### 2.2.7. Stage 5B: Post-Trainer Aggregation Using a Tabular Learner

The second strategy we adopt to the overall positivity of each slide consists in train a classifier to learn the best weights for the indices. More in detail, in this phase, we use the $P_{1}\left(s_{i}\right)$ probabilities not binarized, denoted as $P_{1}^{*}\left(s_{i}\right)$ (i.e., $\frac{1}{T}\sum_{i=1}^{T}p_{i}^{j}$), as well as the set of features $F_{h}\left(s_{i}\right),h\in \left\{0.5,0.6,0.7,0.8,0.9\right\}$ to build the feature matrix detailed in Table 1. Following this approach, we delegate to the algorithm the task of learning how to combine the features in the best possible way. We trained the Tabular learning while using an architecture with three layers composed by respectively 500, 250, and 125 fully connected layers, and we used accuracy as loss function. We did some experiments to include all the $P_{1}\left(s_{i}\right)$, $P_{2}\left(s_{i}\right)$, and $P_{3}\left(s_{i}\right)$ indices in the feature matrix, but we found that the additional indices did not provide a significant increase in the effectiveness scores.

## 3. Results

Table 2 reports the effectiveness of our approach measured on the HEROHE Grand Challenge test set. To the best of our knowledge, there are no other published results (up to current date) that investigate our same problem or use the same test data as we do. For this reason, Table 2 reports the effectiveness scores of our method and the leaderboard of the HEROHE challenge only. Note that the leaderboard has been chosen by the organising committee of the HEROE challenge on the basis of the F1 score alone.

As we can see from the table, if we consider the variants of our approach separately, it is always the case that our system outperforms the leaderboard of the HEROHE challenge, even though different metrics are maximized by different algorithms. The only variant that outperforms the leaderboard of the HEROHE challenge on all the considered metrics is the “All Tiles–Majority Vote” variant.

Turning to compare the different approaches that we propose, we see that it is not always the case that employing the Tabular classifier lead to obtain higher effectiveness scores; more in detail, we see that for precision oriented metrics (i.e., Accuracy and Precision) consider a subset of tiles lead to higher effectiveness scores, and for the metrics affected by recall (i.e., Recall and F1) when considering all of the tiles lead to higher effectiveness scores.

Investigating the most effective approach for each metric separately, we see that our approach leads to obtain an accuracy score of about 0.7 and a precision score of about 0.6, suggesting that our model is able to correctly identify positives in the majority of positive cases. We also see that our model finds almost all positive cases in our dataset (recall of 0.88). When considering the metric used to rank systems in the HEROHE challenge, we see that our model has an F1 score of about 0.7, indicating a good overall balance between precision and recall.

## 4. Discussion and Conclusions

By looking at the results, we can draw different remarks. First, we see that recall (and F1 as result) is maximized when all the tiles are kept in the dataset; this an expected outcome. It is natural that by keeping all of the tiles (i.e., by keeping more data) we obtain a higher recall score. Nevertheless, this behaviour suggests that, when considering the effectiveness metrics all together, it is not straightforward to choose whether to remove or not a subset of tiles, as some tiles might be more informative than others for some metrics, but not for all of them. Finally, this behaviour can be caused not only by the fact that we keep more data, but also by complex interactions between the classification algorithm and some particular feature of the tile. We plan to address this matter in future work. When considering that our problem is a high recall problem, we can say that the best variant of our approach is the ”All Tiles–Majority Vote” one. In other terms, considering our setting we want to find all of the patients that actually are positive for HER2, and we can accept a lower precision if the effort to conduct a clinical test to detect HER2 is not significant, which we believe is a more than reasonable assumption to make: we are willing to spend a little more in clinical testing but be able to save more lives. Summarising the remarks drawn from the results section, we can state that our results indicate that our multi-stage pipeline is indeed effective in detecting HER2. Furthermore, the results indicate that our approach is not over- nor under-dimensioned, given that both the filtering of tiles and the post classifier aggregation done with learners boost the results for some evaluation metric. Finally, we remark that a multi-stage pipeline has the advantage of being easily maintainable, and the accuracy of the single levels can be increased independently.

In this paper, we tackled the problem of classification of the HER2 status on HE slides of invasive breast cancer. Our approach based on a cascade of deep learning classifiers and multi instance learning shows good effectiveness scores for different evaluation metrics. We show that pre-filtering tiles lead to remove potentially noisy tiles and overall leads to an improvement of some effectiveness metrics. We also found that combining different predictors by means of a Tabular classifier is more effective than doing that by the means of a majority voting scores for precision oriented metrics, indicating that, indeed, different predictors need to be weighted and provide signals of different importance for the overall task. However, the overall effectiveness if far from being usable in clinical practice a substitution of the traditional IHC- and ISH-based testing. While this could be due to the intrinsic independency of the information provided by morphology and by HER2 expression, it is also surely conditioned by the relatively low number of slides used for training. In fact, while the overall number of images is high, they correspond to the about 400 cases provided by the Challenge, which are likely too few for Multiple Instance Learning. While no direct comparison is possible about HER2 detection, in other MIL-based related works on digital slides, larger data sets have been used with better results than ours. In particular, in [26] a dataset of about 44,000 slides has been used; although not directly comparable, they reached an AUC of 0.99. In the work of Couture et al. [28] the dataset is closer in size (571 slides) to ours, but also measures of effectiveness, while higher, are not too dissimilar (e.g., 0.80 accuracy for estrogen receptor status, which is a similar but not identical problem as HER2 status). Overall, we think that our work is an important step towards the development of a supervised approach that can serve the purpose of helping medical experts in the delicate task of selecting the appropriate treatment when dealing with clinical cases of breast cancer. For future work, we plan to develop the presented pipeline in order to increase the effectiveness metrics by increasing the impact and the effectiveness of the different stages of the pipeline. One way is to include further case details in the last stage of the pipeline, e.g., the other IHC results, patient data, etc. The other relatively easy enhancement will come from training the cancer classifier on slides acquired with the same setup as the other classifier. In fact, as is the cancer classifier is sub-optimal. In this way, we can provide even more information to the medical experts with the aim of providing a more sound and robust tool that can be practically used in the clinical decision making process.

## Figures and Tables

**Table 1:** Training matrix for the Tabular Learner. $P_{1}^{*}\left(s_{i}\right)$ represents the non binarized $P_{1}\left(s_{i}\right)$ probability. $P(s_{i})$ represents the true positivity for the *i*-th tile (used only for the training phase).

		$P_{1}^{*}$	$F_{0.5}$	$F_{0.6}$	$F_{0.7}$	$F_{0.8}$	$F_{0.9}$		*P*
$s_{1}$		$P_{1}^{*}\left(s_{1}\right)$	$F_{0.5}\left(s_{1}\right)$	$F_{0.6}\left(s_{1}\right)$	$F_{0.7}\left(s_{1}\right)$	$F_{0.8}\left(s_{1}\right)$	$F_{0.9}\left(s_{1}\right)$		$P(s_{1})$
$s_{2}$		$P_{1}^{*}\left(s_{2}\right)$	$F_{0.5}\left(s_{2}\right)$	$F_{0.6}\left(s_{2}\right)$	$F_{0.7}\left(s_{2}\right)$	$F_{0.8}\left(s_{2}\right)$	$F_{0.9}\left(s_{2}\right)$		$P(s_{2})$
⋮		⋮	⋮	⋮	⋮	⋮	⋮		⋮
$s_{i}$		$P_{1}^{*}\left(s_{i}\right)$	$F_{0.5}\left(s_{i}\right)$	$F_{0.6}\left(s_{i}\right)$	$F_{0.7}\left(s_{i}\right)$	$F_{0.8}\left(s_{i}\right)$	$F_{0.9}\left(s_{i}\right)$		$P(s_{i})$
⋮		⋮	⋮	⋮	⋮	⋮	⋮		⋮
$s_{S}$		$P_{1}^{*}\left(s_{S}\right)$	$F_{0.5}\left(s_{S}\right)$	$F_{0.6}\left(s_{S}\right)$	$F_{0.7}\left(s_{S}\right)$	$F_{0.8}\left(s_{S}\right)$	$F_{0.9}\left(s_{S}\right)$		$P(s_{S})$

**Table 2:** Evaluation metrics for our two approaches and the leaderboard of the HEROHE challenge. In bold we highlight the best result obtained for each evaluation metric.

		All Tiles			Subset of Tiles			Best
		Majority Vote	Tabular Classifier		Majority Vote	Tabular Classifier		HEROHE
**Accuracy**		0.687	0.673		0.667	**0.707**		-
**Precision**		0.570	0.560		0.580	**0.603**		0.5682
**Recall**		**0.883**	0.864		0.783	0.797		0.8333
**F1-Score**		**0.693**	0.680		0.667	0.687		0.6757

## Supplementary Materials

The software and trained models are available online at https://github.com/MITEL-UNIUD/HE2HER2/.

## Abbreviations

The following abbreviations are used in this manuscript:

CNN	Convolutional Neural Network
DL	Deep Learning
HER2	Human Epidermal growth factor Receptor 2
MIL	Multiple Instance Learning
ML	Machine Learning
WSI	Whole Slide Image
